# The Book World of Medicine and Science

**Published:** 1903-08-22

**Authors:** 


### THE BOOK WORLD OF MEDICINE AND SCIENCE.

THE IMPERFECTLY DESCENDED TESTIS: ITS ANATOMY, PHYSIOLOGY, AND PATHOLOGY. By W. MCADAM ECCLES, M.S.Lond., F.R.C.S.Eng. (London: Baillière, Tindall and Cox. 1903. Demy 8vo. Pp. 152. Illustrations, 44 plates. Price 7s. 6d. net.)

THIS book embodies the chief facts put forward by Mr. Eccles in the essay which won him the Jacksonian Prize. This of itself is sufficient guarantee of the high value and excellence of the work which the author now publishes in one small volume, with the modest hope that it may prove useful on account of the greater attention that surgeons are now paying to this subject. The essay begins with a description of the normal development and descent of the organ, and it is then shown how imperfect transition affects the structure and functions of the gland. In describing the causes of inflammation of the arrested testis, the malposition renders it more liable to injury, especially from pressure backwards against the pubic bone, which is sure to result in a sharp attack of orchitis. This accident is much more likely to occur in the short tethered testicle, hanging high in the scrotum or retained in the inguinal canal. As other examples of traumatism he mentions the pressure from excessive muscular action, or constant irritation from an improperly applied truss for an associated hernia. The author points out that such orchitis, nearly always terminates in complete atrophy, and even should this not occur after the first attack, a second will almost invariably follow, and lead to certain disorganisation. After an extensive inquiry, he finds he cannot substantiate the teaching which has been handed down, to wit, that the imperfectly descended testis is more prone to suffer from malignant disease than is the organ which is normally placed. The subject of torsion is then carefully considered. The chief predisposing cause, the author states, is partial descent with a freely movable testis. He insists upon the importance of a correct diagnosis of a condition so closely resembling that of strangulated hernia, and he points out the necessity of immediate operative procedures for the removal of the damaged cord and testis. The important relation of hernia, to imperfect descent of the testis, is very fully considered. The author bases his statistics on an analysis of 48,000 cases of hernia in males. He found that imperfect transition occurred in 854, that is in nearly 2 per cent. The monograph is one of the most important which has been written upon the subject of the imperfectly descended testis. To the question, whether a testis arrested from birth, but brought down in early life into the scrotum, will thereby develop and become of procreative value, Mr. Eccles is at present unable to furnish a definite statement. His researches also leave undecided the question, whether the operation of transplantation of the inguinal testis is uniformly or even usually followed by benefit to the organ. As a prophylactic to ward off inflammation, or if associated with hernia he would advocate operative measures, otherwise he does not look to real and lasting benefit with any very strong hopes. He would feel loath to remove the organ, even if useless as a generative gland, so long as there is a chance of its exercising, by a so-called internal secretion, any important influence on the general economy of the body. The book is well written, clearly printed, and easy to read. In bringing the surgery of this subject up to date, the author shows himself to be a possessor of wide and carefully-moderated views, and we warmly recommend the book to the notice of all members of the profession.

FACING THE PHYSICIAN. By M.D. (CANTAB). (London: *The Insurance Agents' News*. Pp. 52. 6d.)

THIS little book is described as "A Handbook for those about to insure, for Insurance Companies, Medical Officers, and Agents." It might equally claim to interest those who are not intending to insure but who ought to be, for it devotes the first of its five chapters to setting forth the advantages of life insurance and designs to show why, from the medical point of view, every man should insure his life. Under the heading of "Advice to Candidates" a few harmless suggestions are made, such as the inadvisability of arriving to undergo the medical examination perfumed with the odours of brandy and tobacco; though no attempt is made, of course, at enabling the candidate to deceive the medical referee into accepting him as a first-class life when he is not really in a fit state to be so accepted. From an educational point of view the booklet has its value, for there can be little doubt that the majority of men do delay taking out insurance policies until such time as they may marry, when they are likely to find the increased premium a heavy addition to the expenses of keeping up a home.

WATER AND AIR: OR NEW THOUGHTS ON OLD SUBJECTS. By J. P. SANDLANDS, M.A., T.C.D., author of "Natural Food," "Sanitation," "The Microbe," "Disease," etc. (The Authors' and Booksellers' Co-operative Alliance, Limited, 151 Strand. Price 4d.)

MR. SANDLANDS seems to be one of those clever people who, having picked up a few bits of scientific knowledge, apply what they have gained in ways which are a little eccentric, and which therefore they believe to be new. His ideas on the subject of air are simply that we need a certain quantity of oxygen in order to be comfortable and well. Given the requisite oxygen, it will not affect our health if unpleasant smells affront our nostrils. As to water, Mr. Sandlands appears to state a paradox when he tells us not to drink water if we wish to be well. But it turns out that the reason for this proscription is simply that we can get all the liquid we need out of fruit. So we can—if we have the fruit. But it does not follow that we can do without liquid of some sort, nor indeed does our author ask this. Indeed, he lays down no new rules of life. The only novelty is his delightful conviction of his own originality.

### BOOKS RECEIVED.

THE SCIENTIFIC PRESS, LIMITED.

"Practical Guide to Surgical Bandaging and Dressings." By Dr. Johnson Smith.

"Nursing Notes on Midwifery and Gynæcology." By Sister Ross.

BAILLIÈRE, TINDALL AND COX.

